# Exercise for premenstrual syndrome: a systematic review and meta-analysis of randomised controlled trials

**DOI:** 10.3399/bjgpopen20X101032

**Published:** 2020-06-10

**Authors:** Emma Pearce, Kate Jolly, Laura L Jones, Gemma Matthewman, Mandana Zanganeh, Amanda Daley

**Affiliations:** 1 Institute of Applied Health Research (IAHR), University of Birmingham, Birmingham, UK; 2 School of Sport, Exercise and Health Sciences, Loughborough University, Loughborough, UK

**Keywords:** premenstrual syndrome, gynaecology, systematic review, meta-analysis

## Abstract

**Background:**

Exercise is recommended as a treatment for premenstrual syndrome (PMS) in clinical guidelines, but this is currently based on poor-quality trial evidence.

**Aim:**

To systematically review the evidence for the effectiveness of exercise as a treatment for PMS.

**Design & setting:**

This systematic review searched eight major databases, including MEDLINE, EMBASE and the Cochrane Central Register of Controlled Trials (CENTRAL), and two trial registries from inception until April 2019.

**Method:**

Randomised controlled trials (RCTs) comparing exercise interventions of a minimum of 8-weeks duration with non-exercise comparator groups in women with PMS were included. Mean change scores for any continuous PMS outcome measure were extracted from eligible trials and standardised mean differences (SMDs) were calculated where possible. Random-effects meta-analysis of the effect of exercise on global PMS symptoms was the primary outcome. Secondary analyses examined the effects of exercise on predetermined clusters of psychological, physical, and behavioural symptoms.

**Results:**

A total of 436 non-duplicate returns were screened, with 15 RCTs eligible for inclusion (*n* = 717). Seven trials contributed data to the primary outcome meta-analysis (*n* = 265); participants randomised to an exercise intervention reported reduced global PMS symptom scores (SMD = -1.08; 95% confidence interval [CI] = -1.88 to -0.29) versus comparator, but with substantial heterogeneity (*I* ^2^ = 87%). Secondary results for psychological (SMD = -1.67; 95% CI = -2.38 to -0.96), physical (SMD = -1.62; 95% CI = -2.41 to -0.83) and behavioural (SMD = -1.94; 95% CI = -2.45 to -1.44) symptom groupings displayed similar findings. Most trials (87%) were considered at high risk of bias.

**Conclusion:**

Based on current evidence, exercise may be an effective treatment for PMS, but some uncertainty remains.

## How this fits in

PMS is a common presentation in primary care settings. Many women are keen to use alternative methods to medical intervention to improve symptoms, but the evidence for these methods is weak. This systematic review and meta-analysis provides further evidence to support the effectiveness of exercise in improving symptoms related to PMS, and will support primary care professionals in recommending treatments for their patients.

## Introduction

PMS describes a broad collection of psychological, physical, and behavioural symptoms, occurring in line with the menstrual cycle.^1–3^ Up to 75% of women may experience symptoms of PMS during their lifetime.^1,4^ Women with PMS frequently present to primary care with acute symptoms, as well as decreased work productivity and relationship difficulties; PMS can also be associated with conditions such as hypertension and depression.^5–7^

Many therapies are recommended for symptom management; for example, both the National Institute for Health and Care Excellence (NICE) and the Royal College of Obstetricians and Gynaecologists (RCOG) suggest exercise as a first-line treatment, alongside medications such as selective serotonin reuptake inhibitors and the combined oral contraceptive pill.^1,8^ The latter two treatments are often effective, but risk side effects such as fatigue, nausea and preclusion of, or potential risks to, pregnancy.^9,10^ Women may also actively prefer alternative therapies.^11^

Nevertheless, exercise is known to increase endorphin levels, to help regulate progesterone and oestrogen synthesis and to encourage the production of endogenous anti-inflammatory chemicals.^12–14^ Exercising also brings other benefits such as improved overall fitness, opportunities to socialise, and the potential for reduction in feelings of depression, all of which may help to moderate the symptom profile in PMS.^2,15,16^

A previous systematic review of this topic found low-level evidence to support the recommendation of exercise for symptom management in PMS.^17^ It is this review that provides the supporting basis for the RCOG recommendation in its PMS guidelines.

### Study aim

This study aimed to provide an updated systematic review and meta-analysis of the evidence examining the effectiveness of exercise as a treatment for PMS. It also undertook to examine the global effect of exercise on PMS symptoms, and the specific effects on each of three symptom domains: psychological, physical, and behavioural. These domains are those specified in the diagnostic questionnaire recommended for use in the UK, the Daily Record of Severity of Problems.^18^

## Method

### Data sources and searches

Cochrane Collaboration guidance on systematic reviews of interventions and PRISMA reporting guidelines were followed throughout.^19,20^ The following databases were searched electronically for eligible trials: MEDLINE, EMBASE, PSYCInfo, CINAHL, AMED, SPORTDiscus, CENTRAL, and clinicaltrials.gov (MEDLINE strategy available at Supplementary figure S1). The World Health Organization International Clinical Trials Registry Platform was searched for trials in progress. The Directory of Open Access Journals was searched; grey literature was sought from OpenGrey; and the authors undertook forward and backward citation searching of included trials.

MeSH and free-text terms relating to the condition of interest (premenstrual syndrome, PMS, premenstrual dysphoric disorder, PMDD, and premenstrual tension) were combined with intervention terms (sport, exercise, physical activity). Scottish Intercollegiate Guideline Network study design limits were applied where possible.^21^ No restrictions were placed by date, language, or country of origin. Searches were undertaken from individual database inception up to April 2018 and refreshed in April 2019; one new article was found at this point, however, the data were already included in the meta-analysis. The authors were contacted where further information was required. Two researchers independently screened all titles and abstracts before reviewing all full-text articles. Any discrepancies were discussed with a third researcher.

### Study selection

Inclusion criteria used were: (a) populations of women reporting regular menstrual cycles (21–35 days) and symptoms of PMS; (b) trials delivering any exercise intervention for a minimum of 8 weeks, including co-interventions such as nutritional supplements, if delivered to both the exercise and non-exercise groups; (c) a non-exercise comparator; (d) any outcome measure quantifying PMS symptoms on a continuous scale; and (e) RCTs. Studies comparing two types of exercise without a non-exercise comparator were excluded.

### Data extraction and quality assessment

Data were independently extracted by two reviewers using an a priori designed and piloted data extraction form. Data from referenced study protocols and author correspondence (where applicable and available) were also used to provide additional information to undertake the quality assessment.

Study quality was independently assessed by three reviewers using quality criteria from the Cochrane Risk of Bias Tool and adapted for inability to blind to group allocation in exercise trials. Criteria concerning selection, performance, detection, attrition, and reporting biases were included and were given equal weighting in the final assessment of overall trial quality. Additional items relating to recall and observer bias, the Hawthorne effect, and contamination bias were also assessed as a group to form the final part of the study quality score.

The quality of the evidence included was assessed by two reviewers using the GRADE (Grading of Recommendations Assessment, Development and Evaluation) methodology for the primary outcome, and all three secondary outcomes; the checklist by Meader *et al* was used to guide this assessment.^22^

### Data synthesis and analysis

SMD and the associated 95% CIs were calculated using standard methods provided by the *Cochrane Handbook for Systematic Reviews*, and guided by a previous systematic review on a related topic.^16,23^ Where studies reported >1 intervention arm testing similar exercise interventions with differing intensities but only one control group, intervention scores were combined to produce one group as per Cochrane Collaboration guidance.^24^

Random-effects meta-analysis was used as heterogeneity within the results was expected.

Between-studies heterogeneity was examined using the *I^2^* statistic, with cut-off points of 25%, 50%, and 75% considered for low, medium, and high heterogeneity, respectively.^25^ Publication bias was assessed narratively by the review team; funnel plots were not constructed as <10 trials contributed data to either the primary or secondary outcome meta-analyses.

All statistical analyses were performed using RevMan 5.3 and IBM SPSS Statistics (version 24).

## Results

### Trial selection

**Figure 1.**
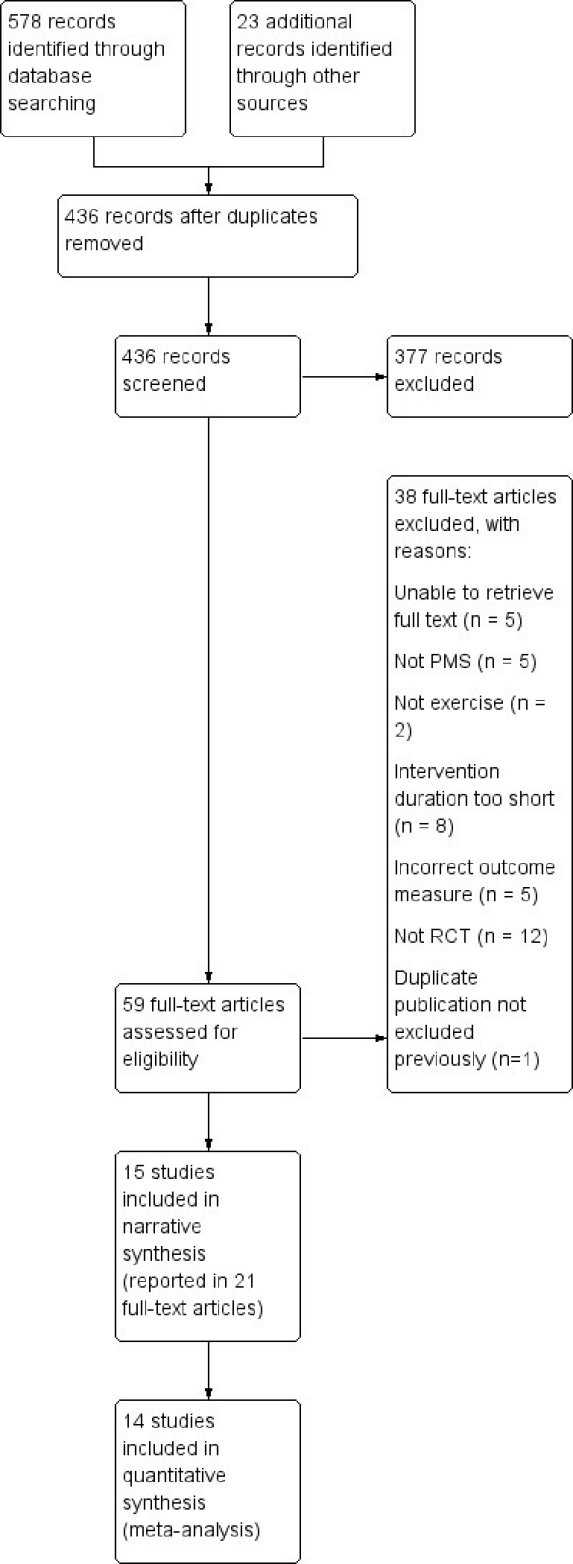
PRISMA flow diagram of included and excluded studies. PMS = premenstural syndrome. RCT = randomised controlled trial.

Following removal of duplicates, 435 titles and abstracts were screened. Of these, 58 full texts were reviewed and 20 reports of 15 individual trials were found to be eligible for inclusion. A further published report of the same trial was found on the PubMed search carried out in April 2019, giving 21 reports of 15 trials in total (Figure 1 and Supplementary Table S1).^26^

### Trial characteristics

A total of 14 published trials^27–40^ and one unpublished PhD thesis^41^ were included in this review. Twelve trials were conducted in Iran^27,29–35,37–40^ (publications for six of these were only available in Persian language), one in Egypt,^28^ and one in South Korea;^36^ the PhD study was conducted in the US.^41^ Sample sizes across the trials ranged from 20–90.

Mean age of participants ranged from 17–34 years across 13 of the trials. Two trials did not publish sufficient data regarding age for this to be extracted.^37,38^ Three trials recruited women from outpatient gynaecology clinics with a clinical diagnosis of PMS,^28,37,40^ and 11 recruited women with symptoms reaching their predefined trial inclusion threshold from community-based locations: six recruited from universities,^30,31,33,35,36,39^ one from a school,^34^ two from the local community,^29,41^ and two from sports clubs.^27,32^ One study did not report the location for recruitment (Table 1).^38^

**Table 1.** Characteristics of included studies.

**Study**	***n***	**Participants**	**Interventions**	**Comparators**	**Outcomes**	**Results**
Abedy *et al*, 2014	30	Sports club attendees, Iran (20–30 years)	Two intervention groups: stretching exercise, resistance exercise; 8-weeks duration, three sessions per week, 60 minutes per session	Usual care	Daily Record of Severity of Problems (not clearly stated within text)	Statistically significant reduction in psychological and behavioural symptoms at 8 weeks regardless of intervention type
Bibi, 1995	50	University and community volunteers, US (18–45 years)	Two intervention groups: moderate intensity aerobic stair stepping, low intensity aerobic stair stepping; 12-weeks duration, three sessions per weeks, 45 minutes per session	Usual care	Daily ratings form	Statistically significant reduction in overall symptoms at 12 weeks regardless of intervention intensity
El-Lithy *et al*, 2015	30	Outpatient clinic attendees, Egypt(16–20 years)	Aerobic exercise with 50 mg vitamin B6 and 1200 mg calcium daily; 12-weeks duration, three sessions per week, 60 minutes per session	50 mg vitamin B6 and 1200 mg calcium only	Modified Premenstrual Syndrome Questionnaire	Statistically significant reduction in overall, psychological, and behavioural symptoms at 12 weeks
Ilka *et al*, 2015	50	Local residents, Iran(20–35 years)	Pilates; 8-weeks duration, three sessions per week, 60 minutes per session, increasing in intensity	Usual care	Premenstrual Symptoms Screening Tool (Iranian)	Statistically significant reduction in symptoms at 8 weeks
Jafarnejad *et al*, 2016(additional duplicates)	70	University Students, Iran (20–40 years)	Home-based aerobic exercise; 8-weeks duration, three sessions per week, 20 minutes per session	Usual care	Daily record of symptoms of premenstrual syndrome	Statistically significant reduction in psychological and physical symptoms at 8 weeks
Kamalifard *et al*, 2017	62	Outpatient clinic attendees, Iran(20–45 years)	Yoga; 10-weeks duration, three sessions per week, 60 minutes per session	Usual care	Premenstrual Symptoms Screening Tool (Iranian)	Statistically significant reduction in psychological, physical, and behavioural symptoms at 10 weeks
Mosallanejad *et al*, 2007	40	University students, Iran (18–25 years)	Aerobic; 8-weeks duration, three sessions per week, 15–45 minutes per session (increasing over the 8 weeks)	Not clearly reported, assumed usual care	Daily Record of Severity of Problems (not clearly stated within text)	Statistically significant reduction in psychological and physical symptoms at 8 weeks
Naeini, 2008	57	Sports club attendees, Iran (18–40 years)	Two intervention groups: 'aerobic', 'physical'; 12-weeks duration, three sessions per week, 20–30 minutes per session	Usual care	Not clearly described	Statistically significant reduction in overall symptoms at 12 weeks regardless of intervention type
Nazemi *et al*, 2015	40(20 included in this review)	University students, Iran (18–25 years)	Group water aerobics; 8-weeks duration, alternate day sessions, 80 minutes per session	Not clearly reported	Short premenstrual assessment form	Statistically significant reduction in symptoms at 8 weeks
Pazoki *et al*, 2016	48	High school students, Iran (16–18 years)	Aerobic exercise; 8-weeks duration, 60 minutes per session. Sessions per week not reported	Usual care (two other groups received fennel extract, and fennel extract with exercise)	Daily Record of Severity of Problems	Statistically significant reduction in overall symptoms at 8 weeks
Samadi *et al*, 2013	40	University students, Iran (18–25 years)	Aerobic exercise; 8-weeks duration, three sessions per week, 60 minutes per session. Hand weights added to one session per week, intensity gradually increased	Usual care	Premenstrual syndrome standard option complaint check list (unvalidated tool)	Statistically significant reduction in overall, psychological, and physical symptoms at 8 weeks
Tonekaboni *et al*, 2012	90	Iran(no age range or recruitment pool given)	Two aerobic exercise intervention groups: high intensity and moderate intensity; 12-weeks duration, three sessions per week, 50 minutes per session. Specialist supervision for all sessions	Usual care	ACOG Daily Symptoms Calendar (unvalidated tool)	Statistically significant reduction in psychological and physical symptoms at 12 weeks
Yang and Kim, 2016	40	Nursing students, Republic of Korea(18–25 years)	Yoga; 12 weeks per duration, one session per week, 60 minutes per session	Usual care (asked to refrain from practising yoga)	Modified Short-Form Menstrual Distress Questionnaire	Statistically significant reduction in overall symptoms at 12 weeks
Yekke Fallah *et al*, 2013	70	University students, Iran (18–32 years)	Two intervention groups: aerobic, fast walking; 12-weeks duration, daily sessions, 30 minutes per session	Usual care	Jack Tips	Reduction in psychological and physical symptoms at 12 weeks regardless of intervention; not statistically significant
Zoodfekr *et al*, 2017	40(20 included in this review)	Hospital clinic patients, Iran (age range not reported)	Aerobic exercise; 8-weeks duration, three sessions per week, 60 minutes per session	Consumed placebo pill (two further intervention groups with different doses of curcumin pills)	Dickerson Questionnaire	Statistically significant reduction in overall symptoms at 8 weeks

### Intervention characteristics

A range of exercise interventions were assessed, including various types of aerobic exercise programmes,^28,30–32,34,35,37–39,41^ yoga regimens,^36,40^ Pilates regimens,^29^ water aerobics programmes,^33^ and stretching and resistance exercise programmes.^27^ When assessing if the exercise was performed in front of a group and/or a researcher, and therefore potentially subject to the Hawthorne bias, eight trials were clearly described as supervised group exercise,^27,31–33,35,36,39,40^ one as a home-based exercise programme to be completed alone,^30^ and the remaining six trials^28,29,34,37,38,41^ did not describe the setting or supervision level in sufficient detail to allow classification. Eleven trials administered the intervention three times per week for the duration of the study period.^27–32,35,37,38,40,41^ No trial delivered the intervention only during the luteal phase. Five trials examined the effects of >1 intensity of exercise intervention against a non-exercise comparator.^27,32,38,39,41^ Intervention duration ranged from 8–12 weeks, and exercise session length from 30 minutes-1 hour. Two trials provided nutritional supplements to all participants, regardless of group allocation.^28,34^

### Risk of bias in included studies and publication bias

**Figure 2.**
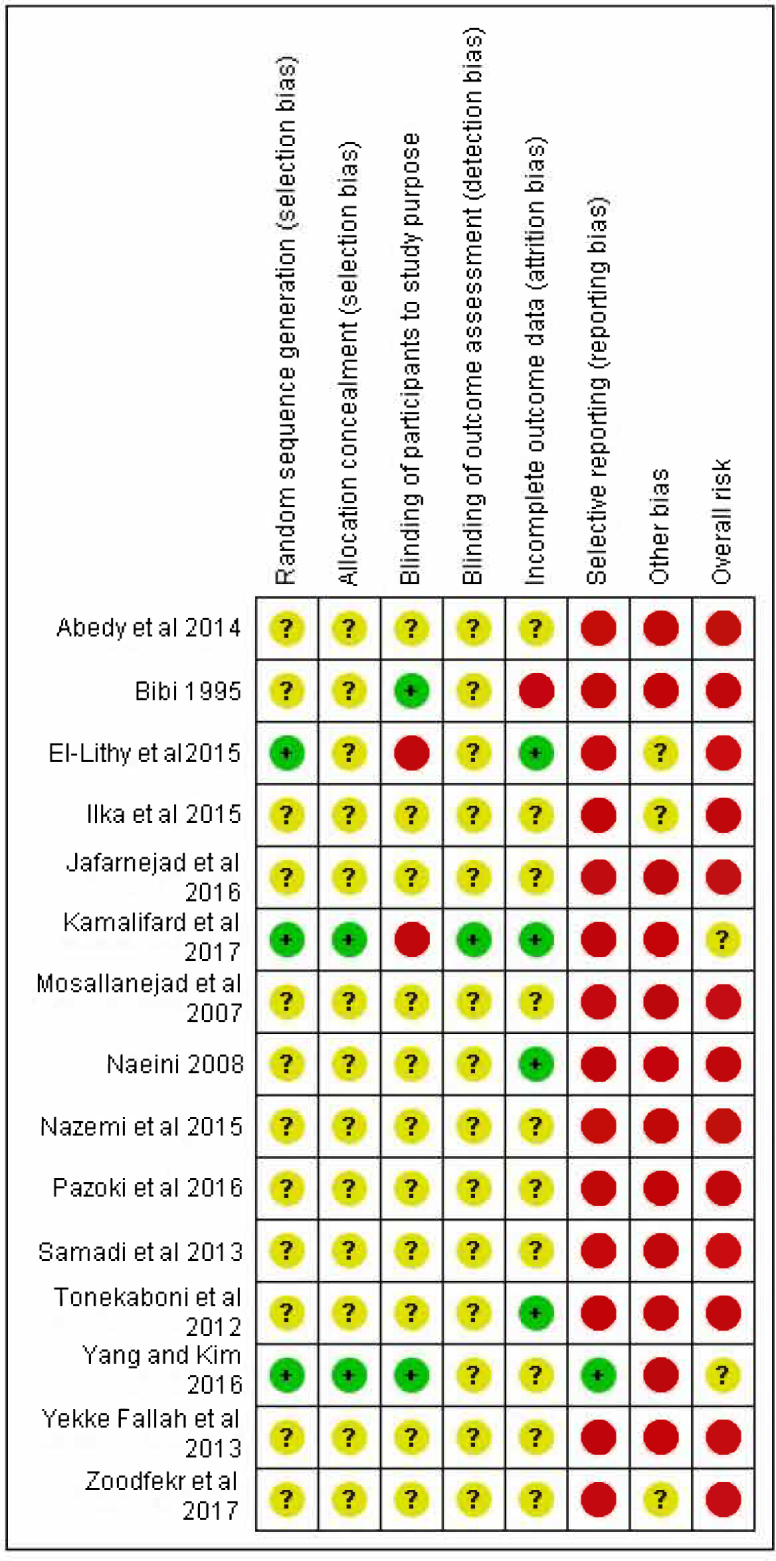
Forest plot for primary outcome: exercise versus control for overall symptom reduction.

Overall, quality of the included trials was low; two^31,39^ were considered unclear (moderate) risk of bias, and the remainder were considered high risk (Figure 2). Identified issues that introduced risk of bias included lack of robust procedures for randomisation, minimal information regarding blinding of participants or assessors, and lack of pre-specified outcomes. Overall assessment of the evidence concluded that there may be a moderate indication of publication bias owing to a lack of negatively reporting trials.

### Study results

All 15 of the included trials reported that exercise significantly decreased symptoms of PMS. Fourteen of the 15 trials reported their data in a format that could be used in meta-analysis of either primary or secondary outcome; Ilka *et al* did not report any standard deviations associated with mean scores or provide adequate data to calculate them, and the author could not be contacted.^29^ The trial conducted by Pazoki *et al* contained four groups, analysed in this review as two pairs: a comparison of exercise intervention versus control, and a second comparison of exercise intervention plus fennel extract versus fennel extract alone.^34^

#### Primary outcome

**Figure 3.**
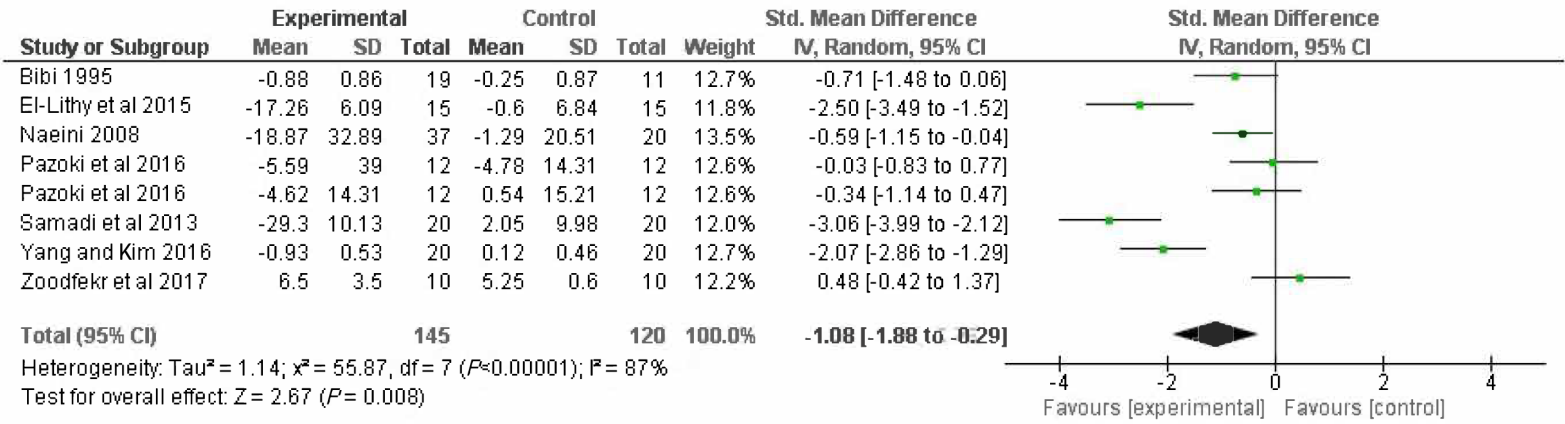
Risk of bias summary. CI = confidence interval. df = degrees of freedom. IV = inverse variance. SD = standard deviation.

Seven of the 15 eligible trials reported global PMS outcome scores (*n* = 265).^28,32,34–37,41^ Meta-analysis of the seven trials reporting on the primary outcome showed a statistically significant reduction in the SMD for participants randomised to exercise interventions compared with comparators (SMD = -1.08; 95% CI = -1.88 to -0.29). Significant heterogeneity existed between trials (*I^2^* = 87%) (Figure 3).

#### Secondary outcomes

Eight of the 15 trials reported data exploring the effect of exercise on psychological symptoms (*n* = 427 participants).^27,28,30,31,35,38–40^ The meta-analysis showed a statistically significant reduction in SMD for psychological symptoms in the exercise group relative to comparators (SMD = -1.67; 95% CI = −2.38 to –0.96). Significant heterogeneity was present (*I^2^* = 89%). Seven of the 15 trials reported data exploring the effect of exercise interventions on physical symptoms of PMS (*n* = 397 participants),^27,30,31,35,38–40^ and meta-analysis showed a statistically significant reduction in SMD for women randomised to exercise interventions compared with comparators (SMD = -1.62; 95% CI = -2.41 to -0.83). Heterogeneity was significant (*I^2^* = 91%). Two of the 15 trials examined the effect of the exercise interventions on behavioural symptoms (*n* = 92 participants),^28,40^ and meta-analysis found a statistically significant reduction in SMD relative to comparators (SMD = -1.94; 95% CI = -2.45 to -1.44) with *I^2^* = 0 (see Supplementary Figures S2-S4).

**Figure 4.**
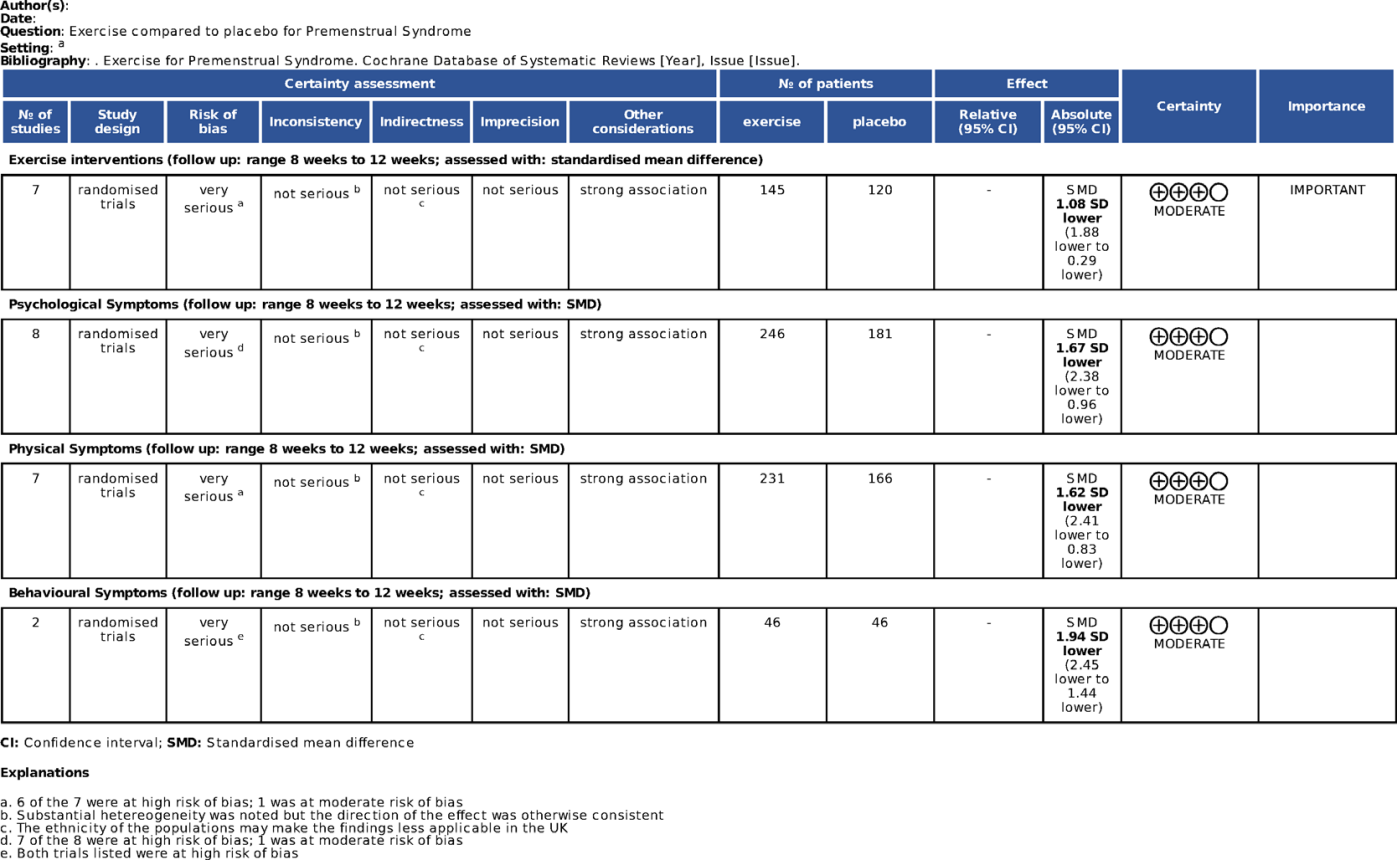
Grading of Recommendations Assessment, Development and Evaluation (GRADE) assessment of overall strength of evidence.

GRADE assessment showed the overall strength of the evidence for all four outcomes to be moderate (Figure 4). Evidence was downgraded from high strength owing to the large number of included trials at high or very high risk of bias.

## Discussion

### Summary

This systematic review provides some further support for clinical guidelines that recommend exercise as an effective treatment for PMS. The secondary analyses conducted also provide new evidence that exercise might be useful in alleviating specific psychological, physical, and behavioural symptoms associated with PMS, as well as assisting with management of the global symptom profile.

### Strengths and limitations

These results should be interpreted cautiously owning to the heterogeneity seen throughout the review. A wide variety of exercise types were included in this review, and the interventions also differed in length and setting. A random-effects meta-analysis model was used to account for this heterogeneity and subgroup analyses were carried out where possible. The initial intention was to further explore any heterogeneity in intervention type; however, too few studies were identified to enable this to be feasible. No studies followed-up women post-intervention so any lasting effects of exercise on symptoms cannot be assessed at this point. Twelve of the 15 included trials were conducted in Iran, where although the prevalence of PMS is similar to that of the UK, the cultural meaning around women’s health and health care more generally may make these results less applicable for some UK women. Most studies recruited small samples, and many did not include a clear description of the interventions assessed, making classifications difficult. Only one of the 15 included trials clearly pre-specified their outcomes,^36^ and different outcome measures were used to assess symptoms across trials.

This review has several strengths. PRISMA guidelines were followed throughout, and the a priori registration of a protocol in PROSPERO utilises all current best-practice guidance for systematic reviews. A comprehensive search strategy, developed in line with similar reviews, was used and multiple electronic databases and grey literature were searched for eligible trials. No language restrictions were made; therefore, it is unlikely that eligible studies were missed for this reason. A native Persian speaker translated all Persian language studies. In only including RCTs in this review, it is hoped the highest quality of evidence currently available on this question has been utilised. The included trials represent women of a range of ages recruited from a variety of clinical and non-clinical settings. For the first time this review descriptively and quantitatively summarises the available evidence on the question of whether exercise is an effective treatment of the symptoms of PMS.

### Comparison with existing literature

The results of this review are in overall agreement with an earlier systematic review by Daley, although that review used broader inclusion criteria, particularly in relation to study design.^17^ The narrative aspect of this updated systematic review examined 15 trials that span a wider range of exercise types and included more participants than the previous review (*n* = 72 versus *n* = 717), but concerns about trial quality remain, along with insufficient reporting detail. The meta-analysis results reported here are consistent with other reviews that have reported exercise to be an effective intervention for primary dysmenorrhoea, and for improving mental and physical health outcomes in the population.^15,42,43^ Exercise is also recommended by the American College of Sports Medicine and NICE to enhance the initial management and advice given on other lifestyle issues known to be associated with PMS, such as obesity and depression.^44–46^

### Implications for research and practice

The results of this systematic review support the recommendation that GPs and other primary healthcare professionals caring for women with PMS should consider exercise as part of their treatment plan. The research recommendations are centred on improving reporting practices, and measures to improve the quality of future trials. It is already strongly suggested that future research in this area should use either the Daily Rating of Severity of Problems^18^ or the Premenstrual Symptoms Screening Tool^47^ alone,: both measure and report global symptom change, as well as symptom cluster results, such as those reported here. By re-administering one of these questionnaires at intervals of 6 months or 1 year, further information relating to the persistence of the positive effects of exercise beyond the end of the trial period could be investigated. If larger sample sizes are used, then subgroup analysis by age, contraceptive use, or other demographic factors may be carried out by future systematic reviewers. Finally, by using the internationally recognised Consolidated Standards of Reporting Trials guidelines,^48^ then the changes suggested above will be clearly communicated to future clinicians and researchers, and can be acted on appropriately.

This systematic review finds that exercise may be an effective treatment for PMS. GPs and other primary healthcare professionals may wish to advise patients that exercise might help reduce their PMS symptoms, but should do so with caution until better-quality evidence becomes available.

## Trial registration number

The review was prospectively registered on PROSPERO: CRD42018088418.
